# Ancestral sequence reconstruction produces thermally stable enzymes with mesophilic enzyme-like catalytic properties

**DOI:** 10.1038/s41598-020-72418-4

**Published:** 2020-09-23

**Authors:** Ryutaro Furukawa, Wakako Toma, Koji Yamazaki, Satoshi Akanuma

**Affiliations:** Faculty of Human Sciences, Waseda University, 2-579-15 Mikajima, Tokorozawa, Saitama 359-1192 Japan

**Keywords:** Protein design, Oxidoreductases

## Abstract

Enzymes have high catalytic efficiency and low environmental impact, and are therefore potentially useful tools for various industrial processes. Crucially, however, natural enzymes do not always have the properties required for specific processes. It may be necessary, therefore, to design, engineer, and evolve enzymes with properties that are not found in natural enzymes. In particular, the creation of enzymes that are thermally stable and catalytically active at low temperature is desirable for processes involving both high and low temperatures. In the current study, we designed two ancestral sequences of 3-isopropylmalate dehydrogenase by an ancestral sequence reconstruction technique based on a phylogenetic analysis of extant homologous amino acid sequences. Genes encoding the designed sequences were artificially synthesized and expressed in *Escherichia coli*. The reconstructed enzymes were found to be slightly more thermally stable than the extant thermophilic homologue from *Thermus thermophilus*. Moreover, they had considerably higher low-temperature catalytic activity as compared with the *T. thermophilus* enzyme. Detailed analyses of their temperature-dependent specific activities and kinetic properties showed that the reconstructed enzymes have catalytic properties similar to those of mesophilic homologues. Collectively, our study demonstrates that ancestral sequence reconstruction can produce a thermally stable enzyme with catalytic properties adapted to low-temperature reactions.

## Introduction

Enzymes, the proteinaceous biocatalysts produced by living organisms, have various superior properties relative to the inorganic catalysts that are often used in the chemical industry^1–3^. The advantages of enzymes include their (1) high rate of catalytic reaction, (2) high enantioselectivity that allows almost 100% discrimination between mirror-image isomers, and (3) low environmental impact owing to their biodegradability. However, enzymes with required properties are not always found in nature because naturally-occurring enzymes have adapted to the host’s specific growth environment and also the competing selection pressures exerted by the cellular environment over the course of evolution^4,5^. For instance, enzymes derived from thermophilic organisms have excellent heat resistance, but often show only very low activity at lower temperatures. By contrast, cold-active enzymes isolated from psychrophilic organisms are often less stable but catalytically more active at low temperatures compared with their mesophilic and thermophilic counterparts^6–11^, although certain cold-active enzymes in nature are also very stable^12,13^. Cold-active enzymes are useful for low temperature processes that preserve thermally instable compounds. In addition, the use of cold-active enzymes in industrial processes sometimes results in lower energy costs^9–11^. For effective use, it is often desirable to create enzymes that have both high thermal stability and strong catalytic activity over a wide range of temperatures, including low and moderate temperatures. At present, however, we do not understand the effects of amino acid substitutions remote from the active site, many of which influence activity and thermostability. Therefore, effective methodology is needed to create thermally stable enzymes with efficient catalytic activity at moderate or even low temperatures without relying on enzyme-design principles^14,15^.

A conventional way to design enzymes with improved properties is the “rational design method,” in which the amino acid sequence is modified based on principles underlying the stability and function of proteins, and detailed tertiary structural information for the target enzyme^16,17^. Nevertheless, modification of an enzyme’s properties remains a challenging task because we still do not have a comprehensive understanding of how proteins physically adapt to high or low temperatures. Directed evolution is another typical way to produce modified enzymes^18,19^. An advantage of this approach is that modified biomolecules with desired properties can be feasibly obtained in the absence of either information on tertiary structure or design principles. Meanwhile, exhaustive investigation of the diversified gene population (library) is needed to select the desired property, a process that often involves considerable time and labor.

In ancestral sequence reconstruction (ASR), inferred ancestral amino acid sequences are created by using homologous amino acid sequences as input information^20–26^. ASR can be a very powerful tool for accessing amino acid substitutions remote from the active site, which influence activity and thermostability^27,28^. This method does not require knowledge about the three-dimensional structure or design principles of the protein. In addition, public databases contain a growing amount of protein sequences, thereby providing a necessary resource for studying ancestral sequence inference. The design method consists of three steps: (1) inference of an ancestral sequence based on a comparison of homologous amino acid sequences; (2) artificial synthesis of a gene encoding the inferred amino acid sequence; and (3) expressing the gene in a host organism such as *Escherichia coli*. Proteins reconstructed in this way are often highly thermostable^29–32^. Moreover, by substituting putative ancestral amino acids into natural enzymes, the thermostability of a natural protein is highly likely to be increased without compromising the catalytic activity^33–37^.

The enzyme 3-isopropylmalate dehydrogenase (IPMDH) is involved in the leucine biosynthetic pathway and encoded by the *leuB* gene. The three-dimensional structure of IPMDH from several species has been determined and, with the exception of enzymes from some hyperthermophilic species^38^, IPMDH exists as a homodimer consisting of two identical subunits^39,40^. As is true for many other enzymes, IPMDH shows a trade-off between thermostability and low-temperature activity^41^. Thermophilic IPMDHs have high thermal stability and low activity at low temperatures, whereas mesophilic enzymes are less thermally stable but highly active at moderate temperatures. IPMDHs are potentially useful for processes involving regeneration of NADH from NAD^+^.

Prior to this study, many mutant enzymes with enhanced catalytic activity at low temperatures had already been obtained from a thermostable IPMDH from the thermophilic bacterium, *Thermus thermophilus* by directed evolution. These studies showed that the catalytic activity at low temperature can be improved without largely affecting the thermal stability^42–44^. Because we wanted to test a protein design approach other than directed evolution, we applied ASR to IPMDH. An ancestral IPMDH corresponding to the last common ancestor of *Bacillus* species has been reconstructed by others^45^. In the present study, we built a phylogenetic tree of extant IPMDHs and their evolutionarily related enzymes, and designed two amino acid sequences of ancestral IPMDH based on the node of the bacterial common ancestor. The catalytic activity and thermostability of the two ancestral IPMDHs were analyzed, showing that these enzymes are highly thermally stable relative to a thermophilic IPMDH and have catalytic properties similar to those of mesophilic IPMDHs. Thus, this work exemplifies how an ancestral design method can create enzymes that simultaneously have thermophilic enzyme-like thermal stability and mesophilic enzyme-like catalytic properties.

## Results

### Inference of ancestral IPMDH sequences

**Figure 1:**
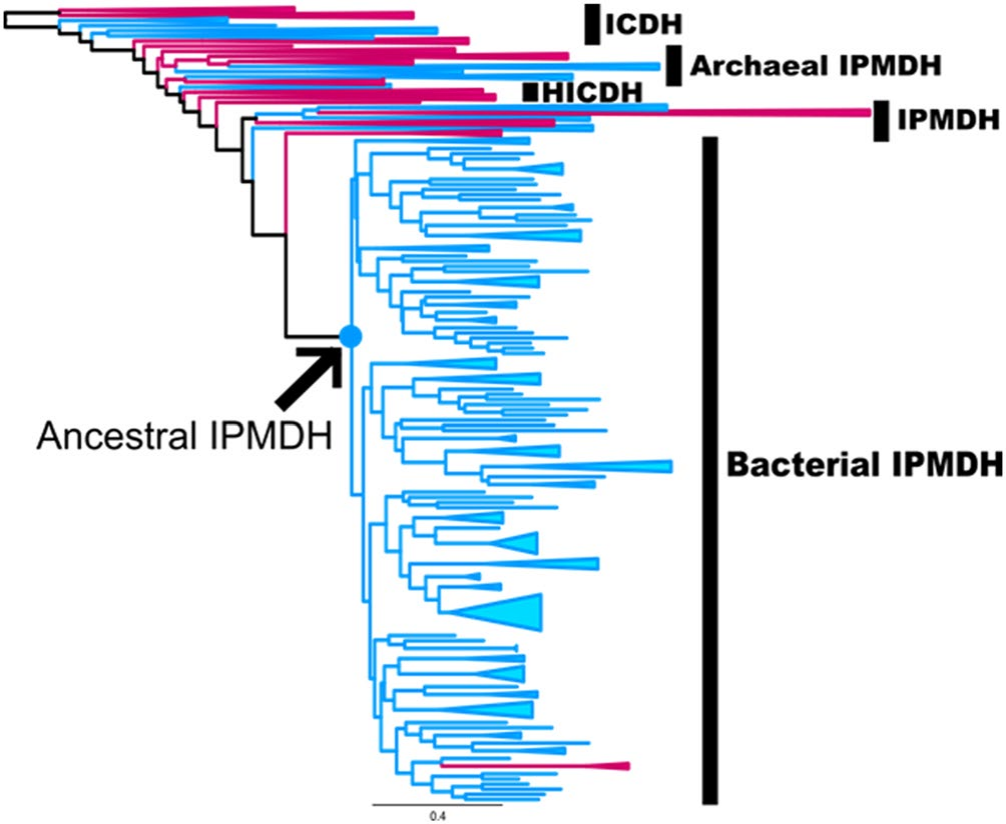
Phylogenetic tree used to infer ancestral IPMDH sequences. The arrow marks the node corresponding to the position of the ancestral IPMDH proteins. Red branches indicate archaeal sequences; blue branches indicate bacterial sequences. For the complete tree, see Fig. S1. The scale bar represents 0.4 substitutions per site.

As the first step in the reconstruction of ancestral sequences of IPMDH, we generated a multiple sequence alignment of 594 IPMDHs and evolutionarily related proteins from extant species. Using IQ-TREE^46^ with a model of rate homogeneity, we built a maximum likelihood tree (Fig. 1; Fig. S1) that was rooted between archaeal isocitrate dehydrogenases and bacterial isocitrate dehydrogenases. The tree shows that IPMDHs and homoisocitrate dehydrogenases diverged from bacterial isocitrate dehydrogenases. Archaeal IPMDHs diverged earlier from bacterial isocitrate dehydrogenases relative to homoisocitrate dehydrogenases and major bacterial IPMDHs, consistent with the previously published IPMDH tree^41^. After diverging from archaeal IPMDHs, homoisocitrate dehydrogenases diverged multiple times. The complex group of IPMDHs subsequently diverged from homoisocitrate dehydrogenases, and included archaeal IPMDHs, bacterial IPMDHs, and tartrate dehydrogenase. Lastly, the bacterial IPMDHs group diverged to form major bacterial IPMDHs and thermophilic bacterial IPMDHs.

**Figure 2:**
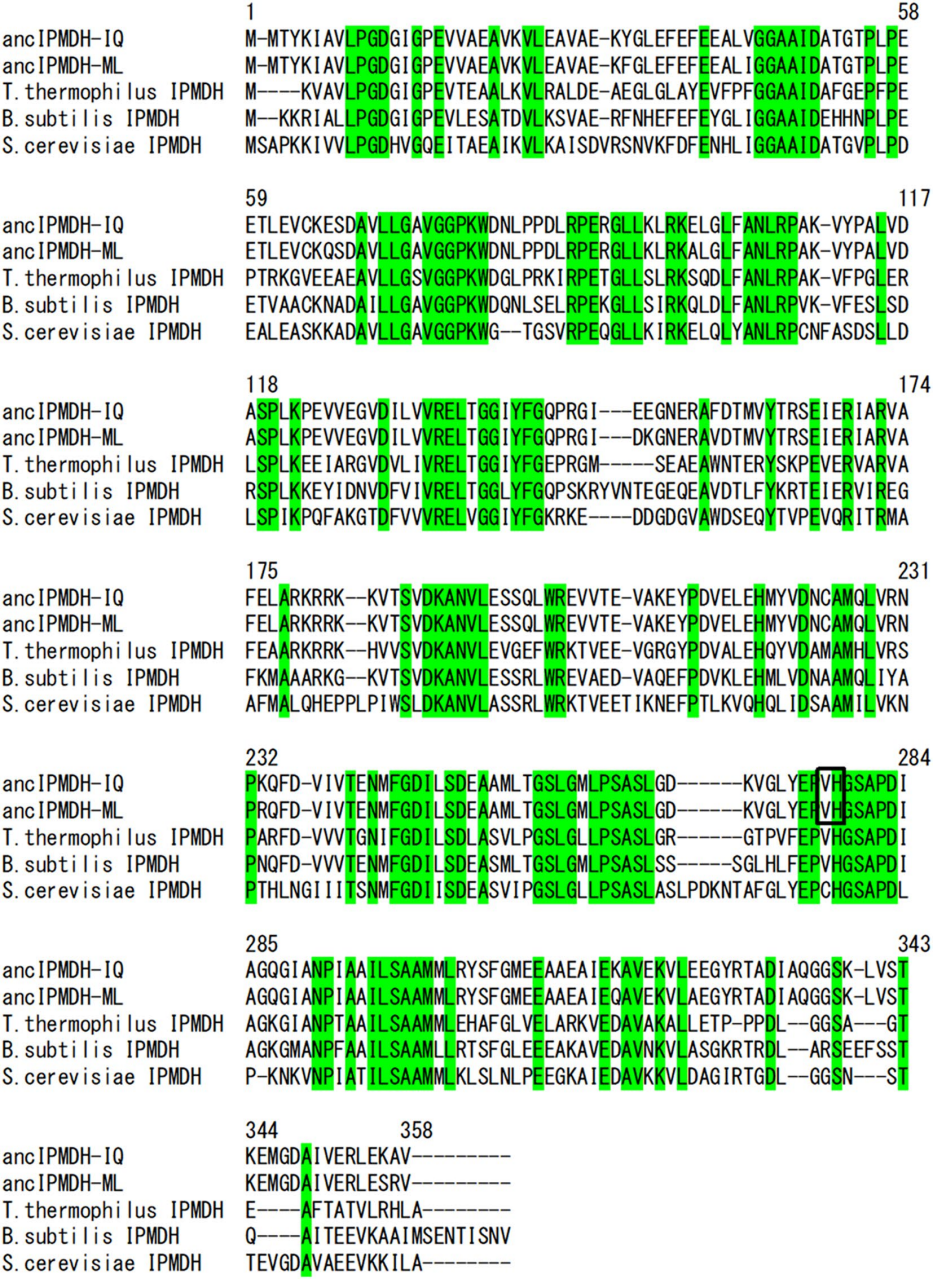
Multiple sequence alignment of ancIPMDH-IQ, ancIPMDH-ML, *T. thermophilus* IPMDH, *B. subtilis* IPMDH, and *S. cerevisiae* IPMDH. Numbers above the sequences are those of the ancestral enzymes. Residues conserved among the five sequences are highlighted in green. V277 and H278, the two residues mutated in the ancestral sequences, producing ancIPMDH-IQ-VAHG and ancIPMDH-ML-VAHG, are boxed.

Two different ancestral sequences at the node corresponding to the last common ancestor of major bacterial IPMDHs were inferred from the tree by using IQ-TREE^46^ and CodeML in PAML^47^, and named ancIPMDH-IQ and ancIPMDH-ML, respectively (Fig. 2; Table S1). The two ancestral IPMDHs share 96.4% amino acid sequence identity. Comparison of the amino acid sequences of ancIPMDH-IQ and ancIPMDH-ML with that of *T. thermophilus* IPMDH showed that, respectively, 64.0% and 63.4% of 322 aligned residues are identical (Fig. 2). When the sequences of ancIPMDH-IQ and ancIPMDH-ML were compared with that of *Bacillus subtilis* IPMDH, 63.7% and 62.9% of 361 aligned residues are identical, respectively (Fig. 2). The ancestral IPMDH sequences have 60.3%, 60.7% and 59.8% amino acid sequence identity with psychrophilic IPMDHs from *Shewanella violacea*, *Pseudoalteromonas haloplanktis* and *Psychrobacter cryohalolentis*, respectively. Genes encoding the ancIPMDH-IQ and ancIPMDH-ML amino acid sequences were artificially synthesized and expressed in *E. coli* using a Novagen pET protein expression system, and the two ancestral enzymes were purified by successive chromatography with HiTrap-Butyl and ResourceQ columns and then characterized.

### Oligomeric structure of the ancestral IPMDHs

**Figure 3:**
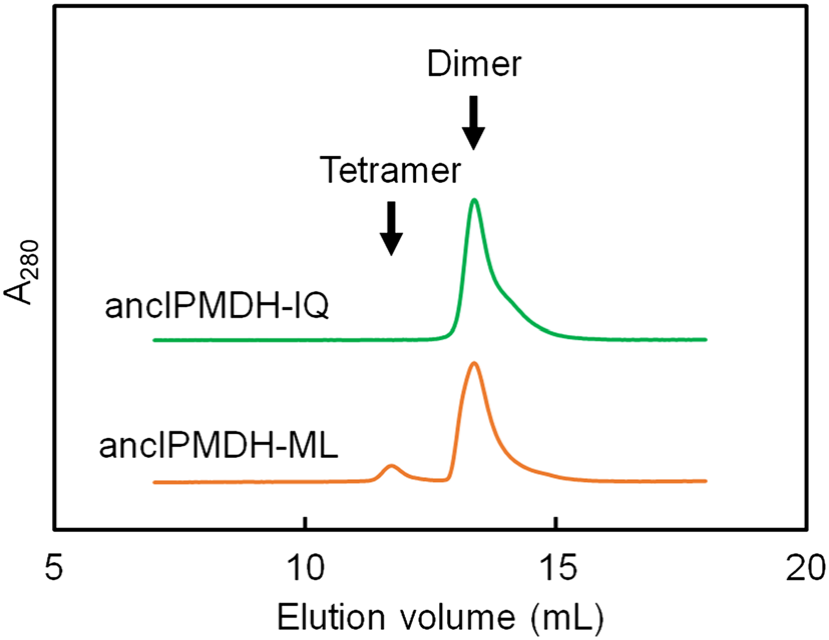
Analytical gel filtration using Superdex200 Increase resin. Proteins were applied at 5 μM in 20 mM potassium phosphate, pH 7.6, 150 mM KCl, and 1 mM EDTA with a flow rate of 0.7 ml/min. Elution positions corresponding to the dimer and hexamer are indicated. A_280_, absorbance at 280 nm.

Although most naturally-occurring IPMDHs exist as homodimers consisting of two identical subunits^39,40^, homotetrameric IPMDHs are observed in some hyperthermophilic species^38^. We therefore investigated the oligomeric state of the ancestral enzymes by using analytical gel filtration (Fig. 3). The elution profile showed that ancIPMDH-IQ migrates as a single molecular species with a retention volume corresponding to the molecular weight expected for a dimer. The elution profile of ancIPMDH-ML suggested that this enzyme is predominantly a dimer, but a small elution peak corresponding to a tetramer was also observed.

### Thermal stability

**Figure 4:**
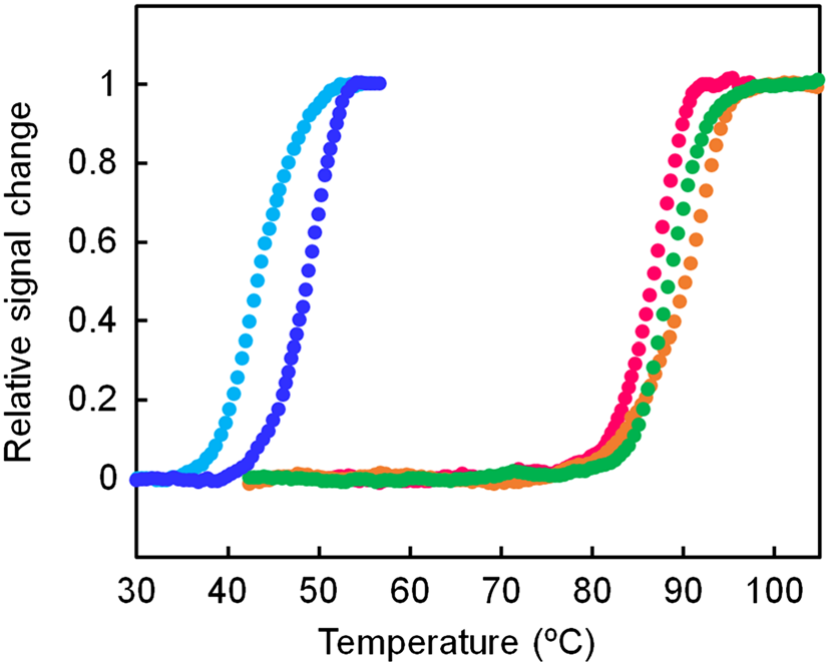
Thermal melting curves of the ancestral and extant IPMDHs. The change in ellipticity at 222 nm was monitored as a function of temperature. The scan rate was 1.0 °C/min. The samples comprised 5.0 μM protein in 20 mM potassium phosphate (pH 7.6), 0.5 mM EDTA. Each experiment was conducted in duplicate with identical melting profiles within experimental error. The plots were normalized with respect to the baseline of the native and denatured states. Green, ancIPMDH-IQ; orange, ancIPMDH-ML; magenta, *T. thermophilus* PMDH; blue, *B. subtilis* IPMDH; cyan, *S. cerevisiae* IPMDH.

The thermal stability of ancIPMDH-IQ and ancIPMDH-ML was estimated by circular dichroism (CD) measurement. We monitored the change in ellipticity at 222 nm for a 5 μM protein solution in 20 mM potassium phosphate buffer (pH 7.6), 1 mM EDTA. The temperature-induced unfolding curves shown in Fig. 4 were normalized by assuming a linear temperature dependence between the baselines of the fully native and the fully unfolded states. The unfolding curves of IPMDHs from the thermophilic bacterium *T. thermophilus*, the mesophilic bacterium *B. subtilis*, and the eukaryotic microorganism *Saccharomyces cerevisiae*, obtained in previous studies^41^, were included in Fig. 4 for comparison.

The unfolding mid-point temperature (*T*_*m*_) was used to compare the thermal stability of the proteins (Table 1). The *T*_m_ values of ancIPMDH-IQ and ancIPMDH-ML were 88 and 90 °C, respectively, which were slightly higher than that (86 °C) of *T. thermophilus* IPMDH. Figure 4 also shows that the thermal stabilities of the two ancestral enzymes were much higher than those of extant mesophilic IPMDHs from *B. subtilis* (*T*_m_ = 48 °C) and *S. cerevisiae* (*T*_m_ = 43 °C).

**Table 1:** Unfolding midpoint temperatures, optimum catalytic temperatures and specific activities of ancIPMDH-IQ, ancIPMDH-ML and their mutant enzymes.

Enzyme	*T*_m_ (°C)^a^	*T*_opt_ (°C)^b^	Specific activity (U/mg)^c^	
			25 °C	70 °C
ancIPMDH-IQ	88	75	2.76 ± 0.02	38.5 ± 0.5
ancIPMDH-ML	90	77	1.87 ± 0.04	39.1 ± 0.1
ancIPMDH-IQ-VAHG	83	67	3.56 ± 0.13	29.6 ± 0.5
ancIPMDH-ML-VAHG	85	68	2.74 ± 0.10	22.4 ± 0.4

^a^*T*_m_ values were estimated from the data shown in Figs. 2 and S5.

^b^*T*_opt_ values are the optimum temperatures for catalytic activity estimated from the data shown in Figs. 5b and 6b.

^c^Average values and standard errors were calculated from three independent measurements.

### Temperature dependence of specific activity

**Figure 5:**
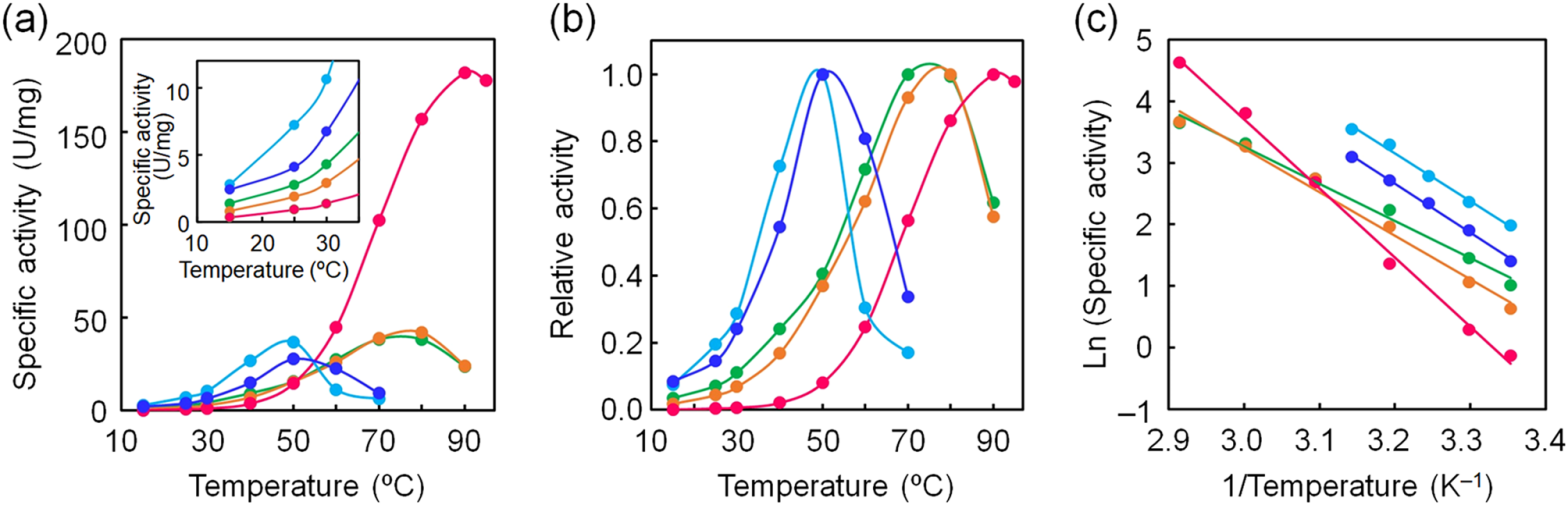
Specific activities of the ancestral and extant IPMDHs. (**a**) Plot of specific activity as a function of temperature. The assay solution was composed of 50 mM HEPES (pH 8.0), 100 mM KCl, 5 mM MgCl_2_, 0.2 mM D-3-IPM, 5 mM NAD^+^, and 0.1–1.0 μM protein. Each value is the average of three measurements. The specific activities of both ancIPMDH-IQ and ancIPMDH-ML were greater than that of *T. thermophilus* enzymes by a factor of more than three and two, respectively, below 30 °C (inset). (**b**) Plot of relative activity as a function of temperature. Shown are the relative values of specific activity at various temperatures compared with the activity at the optimal temperature for each enzyme. (**c**) Arrhenius plot of the specific activities of the ancestral and extant IPMDHs. Activation energy (*E*_a_) was calculated from the slope of each plot. *E*_a_: ancIPMDH-IQ, 50 kJ/mol; ancIPMDH-ML, 59 kJ/mol; *T. thermophilus* PMDH, 93 kJ/mol; *B. subtilis* IPMDH, 66 kJ/mol; *S. cerevisiae* IPMDH, 64 kJ/mol. Green, ancIPMDH-IQ; orange, ancIPMDH-ML; magenta, *T. thermophilus* PMDH; blue, *B. subtilis* IPMDH; cyan, *S. cerevisiae* IPMDH.

We measured the enzymatic activity of the ancestral IPMDHs by assessing the oxidative decarboxylation of D-3-isopropylmalate (D-3-IPM) to produce 2-oxoisocaproate, using NAD^+^ as the electron acceptor. Figure 5a shows the temperature dependence of the specific activities of the ancestral IPMDHs, as well as those of IPMDHs from thermophilic *T. thermophilus* and two mesophilic species (*B. subtilis* and *S. cerevisiae*) for comparison. Although the ancestral IPMDHs were slightly less catalytically active relative to the mesophilic IPMDHs, they were more active than the thermophilic IPMDH at temperatures ranging from 25 to 50 °C. Remarkably, the specific activities of ancIPMDH-IQ and ancIPMDH-ML were, respectively, 3.1- and 2.1-fold greater than that of *T. thermophilus* IPMDH at 25 °C (Table 1). At 70 °C, the specificity activities of the ancestral IPMDHs were greater than those of the mesophilic IPMDHs but smaller than that of the thermophilic enzyme.

Figure 5b shows the relative activities at various temperatures compared with the activity at the optimal temperature for each enzyme. Generally, cold-adapted enzymes maintain ~ 10–20% of their maximum activity at low temperatures and their thermal inactivation precedes the thermal unfolding^48^. The thermophilic *T. thermophilus* IPMDH functioned optimally at 90 °C under the reaction condition employed and the activity was reduced to 0.5% of the maximum activity at 25 °C; by contrast, the mesophilic IPMDHs from *B. subtilis* and *S. cerevisiae* functioned optimally at 50 °C and maintained, respectively, 15% and 20% of their maximum activities at 25 °C. ancIPMDH-IQ and ancIPMDH-ML functioned optimally at 70–80 °C (Table 1) and maintained, respectively, 7% and 4% of their maximum activities at 25 °C. Thus, the ancestral IPMDHs were better adapted to low reaction temperatures compared to the thermophilic IPMDH as judged by the temperature profiles of the relative activities.

The activation energy (*E*_a_) for the specific activity of the ancestral IPMDHs, as well as *T. thermophilus*, *B. subtilis* and *S. cerevisiae* IPMDHs, was calculated from the slope of the Arrhenius plot for the temperature range 25–70 °C (Fig. 5c). A smaller *E*_a_ value indicates that the reaction rate has less temperature dependency, and is thus at least partially associated with the higher catalytic activity of a mesophilic enzyme at low temperature relative to its thermophilic counterpart. Indeed, the mesophilic IPMDHs had smaller *E*_a_ values (*B. subtilis* IPMDH, 66 kJ/mol; *S. cerevisiae* IPMDH, 64 kJ/mol) as compared with the *T. thermophilus* enzyme (93 kJ/mol). The ancestral IPMDHs had further reduced *E*_a_ values (ancIPMDH-IQ, 50 kJ/mol; ancIPMDH-ML, 59 kJ/mol) as compared with the mesophilic IPMDHs. Overall, the temperature dependency of the specific activity of the ancestral IPMDHs resembles that of the mesophilic IPMDHs more closely than that of the thermophilic enzyme.

### Kinetic parameters

We estimated the kinetic parameters of the catalytic activity of the ancestral IPMDHs from steady-state experimental data obtained at 25, 40 and 70 °C (Table 2). We previously reported that changes in the specific activity of IPMDH correlate mainly to the change in *K*_m_ for the coenzyme NAD^+^ and not to the change in *K*_m_ for the substrate (D-3-IPM)^44^. In addition, amino acid substitutions that modulate the coenzyme-binding pocket affect the temperature dependency of the catalytic activity of *T. thermophilus* IPMDH.

**Table 2:** Kinetic constants of the ancestral IPMDHs, their mutants, and extant IPMDHs.

Enzyme	*K*_m_^D-3-IPM^ (μM)^a^	*K*_m_^NAD^ (μM)^a^	*k*_cat_ (s^−1^)^a^
25 °C			
ancIPMDH-IQ	n.d.^b^	45 ± 6	1.7 ± 0.1
ancIPMDH-ML	n.d.^b^	37 ± 4	1.2 ± 0.0
ancIPMDH-IQ-VAHG	n.d.^b^	77 ± 4	2.0 ± 0.0
ancIPMDH-ML-VAHG	n.d.^b^	33 ± 2	1.6 ± 0.0
*T. thermophilus* IPMDH	n.d.^b^	2.2 ± 0.3	0.37 ± 0.01
*B. subtilis* IPMDH	n.d.^b^	110 ± 10	2.8 ± 0.1
*S. cerevisiae* IPMDH	n.d.^b^	130 ± 10	5.1 ± 0.1
40 °C			
ancIPMDH-IQ	5.3 ± 0.2	67 ± 7	4.6 ± 0.1
ancIPMDH-ML	4.7 ± 0.4	54 ± 6	4.3 ± 0.1
ancIPMDH-IQ-VAHG	5.0 ± 0.2	230 ± 12	8.5 ± 0.1
ancIPMDH-ML-VAHG	5.0 ± 0.3	240 ± 40	5.0 ± 0.3
*T. thermophilus* IPMDH	2.3 ± 0.8	12 ± 1	2.4 ± 0.1
*B. subtilis* IPMDH	0.78 ± 0.11	420 ± 20	9.4 ± 0.2
*S. cerevisiae* IPMDH	4.5 ± 0.7	650 ± 70	20 ± 1
70 °C			
ancIPMDH-IQ	10 ± 2	960 ± 50	22 ± 0
ancIPMDH-ML	10 ± 2	730 ± 80	22 ± 1
ancIPMDH-IQ-VAHG	13 ± 3	4,100 ± 500	34 ± 2
ancIPMDH-ML-VAHG	11 ± 2	3,300 ± 400	24 ± 1
*T. thermophilus* IPMDH	3.7 ± 1.4	210 ± 10	79 ± 3

^a^*K*_m_ and *k*_cat_ were calculated from steady-state experiments performed at 25, 40, or 70 °C with an assay buffer containing 50 mM HEPES (pH 8.0), 100 mM KCl, 5 mM MgCl_2_, and various concentrations of D-3-IPM and NAD^+^. The values and standard errors were obtained by nonlinear least-square fitting of the steady-state velocity to the Michaelis–Menten equation using the Enzyme Kinetics module of SigmaPlot version 13.0, from Systat Software, Inc., San Jose California USA, www.systatsoftware.com.

^b^*K*_m_ values for D-3-IPM at 25 °C could not be determined because appropriately designed steady-state kinetic experiments would include reactions that use very small amounts of D-3-IPM, which would be consumed quickly and preclude accurate velocity measurements.

Indeed, although the *K*_m_^D-3-IPM^ values of the ancestral IPMDHs were less favorable than those of the thermophilic and mesophilic IPMDHs at 40 °C, the variation observed among the *K*_m_^D-3-IPM^ values was smaller than that observed among the *K*_m_^NAD^ values of the ancestral and extant IPMDHs. In addition, the ancestral IPMDHs had significantly worse *K*_m_^NAD^ values as compared with the *T. thermophilus* enzyme at 25, 40 and 70 °C; however, the *k*_cat_ values of ancIPMDH-IQ and ancIPMDH-ML were, respectively 4.6- and 3.2-fold greater than that of *T. thermophilus* IPMDH at 25 °C. At 40 °C, the ancestral IPMDHs also showed greater *k*_cat_ values relative to the *T. thermophilus* enzyme. At 70 °C, the *k*_cat_ values of the ancestral IPMDHs were only 28% of the *k*_cat_ value of *T. thermophilus* IPMDH. It has been reported that adaptation of an enzyme to low temperature is accompanied by less favorable *K*_m_ and improved *k*_cat_ values^49–51^. Therefore, the catalytic properties of the ancestral IPMDHs are better adapted to low temperature as compared with the thermophilic enzyme.

In a comparison of the kinetic parameters of the ancestral IPMDHs with those of the mesophilic enzymes, the ancestral enzymes had smaller *k*_cat_ values, but had improved *K*_m_^NAD^ values at 25 and 40 °C.

### Attempt at further adaptation of ancestral IPMDHs to low-temperature reaction

We previously created *T. thermophilus* IPMDH mutants in which one or a few amino acid(s) located within a 12-Å distance from the active site was/were replaced by the residue(s) found at the same position(s) in the amino acid sequence of the *E. coli* IPMDH^41^. One of the mutants, which contained two amino acid substitutions, Val272 → Ala and His273 → Gly, had 7.6-fold greater specific activity at 25 °C, and a *T*_m_ value only 2 °C lower than that of the wild-type enzyme^41^. Both residues are located near the active site of the thermophilic wild-type enzyme: the side chain of Val272 is directly opposite the active site, whereas the side chain of His273 interacts with the adenine of NAD^+^. Because these residues are conserved in the amino acid sequences of ancIPMDH-IQ and ancIPMDH-ML (Val277 and His278; Fig. 2), we replaced Val277 and His278 with Ala and Gly, respectively, in both ancestral IMPDHs by site-directed mutagenesis to produce ancIPMDH-IQ-VAHG and ancIPMDH-ML-VAHG. The genes encoding the mutants were then expressed in *E. coli*, and the proteins were purified to homogeneity.

**Figure 6:**
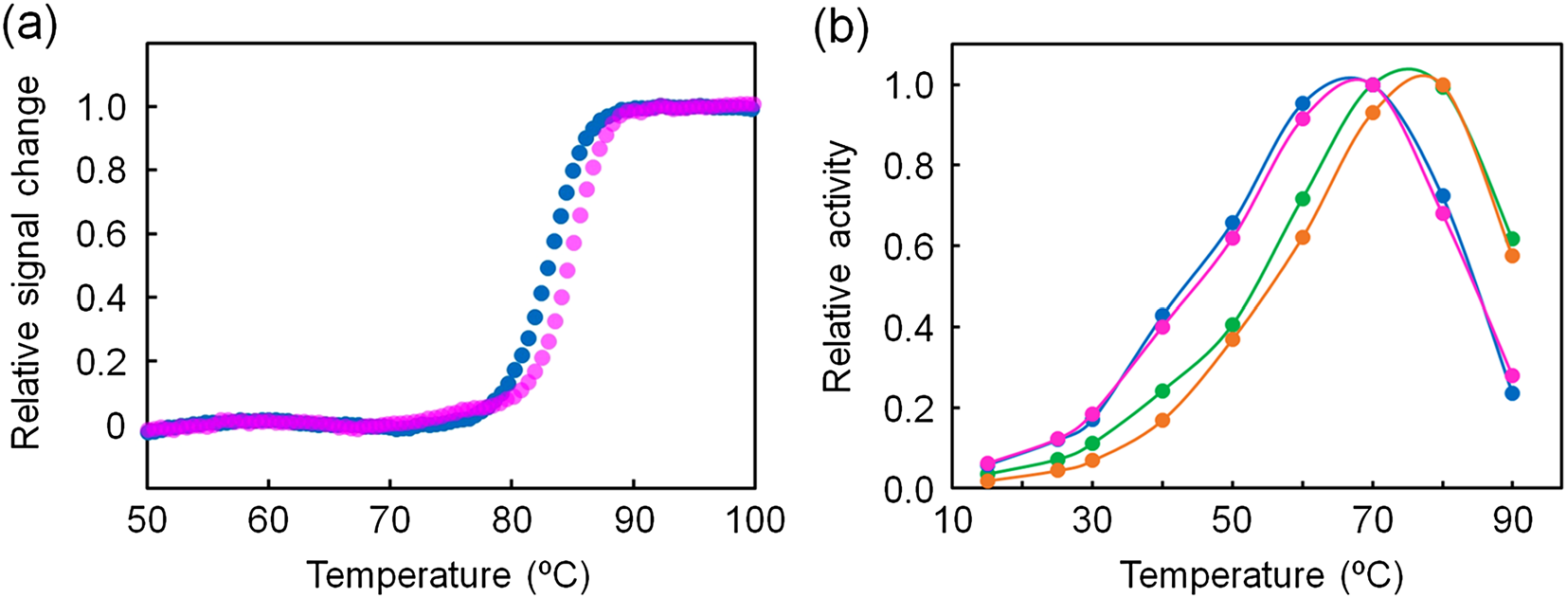
Characterization of the mutants of the ancestral IPMDHs. (**a**) Thermal melting curves of ancIPMDH-IQ-VAHG (blue) and ancIPMDH-ML-VAHG (magenta). The change in ellipticity at 222 nm was monitored as a function of temperature. The scan rate was 1.0 °C/min. The samples were comprised of 5.0 μM protein in 20 mM potassium phosphate (pH 7.6), 0.5 mM EDTA. Each experiment was conducted in duplicate with identical melting profiles within experimental error. The plots were normalized with respect to the baseline of the native and denatured states. (**b**) Plot of relative activities of ancIPMDH-IQ (green), ancIPMDH-ML (orange), ancIPMDH-IQ-VAHG (blue) and ancIPMDH-ML-VAHG (magenta) as a function of temperature. Shown are the relative values of specific activity at various temperatures compared with the activity at the optimal temperature for each enzyme.

The structural properties of ancestral IPMDHs and their mutants were investigated by using CD spectroscopy to assess their secondary structure (Fig. S2). The far-UV CD spectra of ancIPMDH-IQ and ancIPMDH-ML as well as those of ancIPMDH-IQ-VAHG and ancIPMDH-ML-VAHG were quite similar, indicating that they have almost same amounts and types of secondary structure. Thermal unfolding experiment of the mutants showed that the *T*_m_ values of ancIPMDH-IQ-VAHG and ancIPMDH-ML-VAHG were 83 and 85 °C, respectively (Table 1; Fig. 6a). Thus, the *T*_m_ values of the mutants were slightly lower than those of the parent ancestral enzymes, but still much higher than those of the mesophilic *B. subtilis* and *S. cerevisiae* IPMDHs.

Enzymatic activity measurements indicated that the mutants had slightly improved specific activities at 25 °C (Table 1): the activities at 25 °C of ancIPMDH-IQ-VAHG (3.6 U/mg) and ancIPMDH-ML-VAHG (2.7 U/mg) were, respectively, 1.3- and 1.5-fold greater than those of ancIPMDH-IQ (2.8 U/mg) and ancIPMDH-ML (1.9 U/mg). Therefore, the extent to which the amino acid substitutions enhanced the specific activity at low temperature was substantially smaller than when the amino acid substitutions were introduced into *T. thermophilus* IPMDH.

The relative activities of ancIPMDH-IQ-VAHG and ancIPMDH-ML-VAHG as a function of temperature were compared with those of ancIPMDH-IQ and ancIPMDH-ML (Fig. 6b; Table 1), and showed that the optimal temperatures for ancIPMDH-IQ-VAHG and ancIPMDH-ML-VAHG shifted to low temperatures. In addition, ancIPMDH-IQ-VAHG and ancIPMDH-ML-VAHG maintained greater relative activities at 15–60 °C. Therefore, the mutants were further adapted to low reaction temperatures compared to their parent ancestral IPMDHs.

The kinetic parameters of ancIPMDH-IQ-VAHG and ancIPMDH-ML-VAHG were also estimated by using steady-state kinetic data obtained at 25, 40 and 70 °C (Table 2). The *K*_m_^D-3-IPM^ values of the two mutants at 40 and 70 °C were, within the margin of error, the same as those of the parent ancestral IPMDHs. Although the *K*_m_^NAD^ values of the mutants at 25 °C were similar to or slightly worse than those of the ancestral IPMDHs, the mutants had significantly worse *K*_m_^NAD^ values as compared with the ancestral enzyme at 70 °C. Therefore, the substitutions unfavorably affected interaction with the coenzyme to a larger extent at higher temperatures. Lastly, the two amino acid substitutions only slightly improved the *k*_cat_ values of the ancestral IPMDHs at 25, 40 and 70 °C (Table 2). When the Val272 → Ala and His273 → Gly substitutions were introduced into *T. thermophilus* IPMDH, by contrast, the *k*_cat_ value was substantially improved by a factor of 9.5 at 25 °C^41^. Taken together, these data show that identical amino acid substitutions may not have the same effect in different proteins even if the proteins are homologous.

## Discussion

Reconstruction of ancestral sequences based on a comparison of extant homologous protein sequences is a way to infer ancient protein sequences that were plausibly present in extinct species^20–23^. In combination with an empirical analysis of the reconstructed proteins, this method has been previously used to characterize the physical properties of ancestral proteins, including thermal stability and substrate specificity^29,30,52–58^. The sequence reconstruction approach has also been used to investigate the evolution of protein folds^59^ and to identify key amino acid residues in a metabolic enzyme complex^60^. It also allows us to deduce information on the history of life on Earth and to estimate long-term changes in the environment of the biosphere^30,58,61^.

ASR may help to engineer proteins and enzymes that are potentially useful for industrial processes because the ancestral design may create enzymes with desired traits that are not found in naturally-occurring enzymes^62^. For example, Ito and coworkers engineered an ancestral L-amino acid oxidase by using the phylogeny-based method^63^. Unlike naturally-occurring L-amino acid oxidases, the ancestral oxidase had broad substrate selectivity and high productivity via a prokaryotic heterologous expression system. As a result, the ancestral oxidase will be potentially useful for yielding pure d-amino acids from D,L-mixtures by enzymatic deracemization. Gillam and coworkers also engineered highly functional thermostable vertebrate CYP3 P450 ancestor by ASR^64^.

Herein, we have described the production of enzymes that have thermophilic enzyme-like thermal stability and mesophilic enzyme-like catalytic properties by ASR. Although the relationship between thermal stability and low-temperature catalytic activity varies in naturally occurring enzymes, incompatibility between thermal stability and low-temperature activity are sometimes found^41,50,65,66^. The incompatibility is often problematic in the industrial use of enzymes^6,67^. For example, the high catalytic activities of cold-adapted enzymes at low temperatures are often suitable for food and beverage processing, but the concurrent thermolability of the cold-adapted enzymes often limits their use in such applications because the enzymes may be inactivated by small changes in temperature^14,68^. Thus, the creation of enzymes with great thermal stability and catalytic efficiency at moderate temperature is highly desirable. Several examples have demonstrated that the incompatibility between thermal stability and moderate or even low temperature activity can be avoided^12,13,44,69,70^. The GH42 cold-active β-galactosidase from the psychrophilic bacterium *Marinomonas* ef1 combines cold activity with unusual thermostability^71^. Moreover, phylogenetic analyses indicate a close relationship with thermophilic β-galactosidases, suggesting that this enzyme evolved from a thermostable scaffold. Those examples encouraged us to create enzymes that simultaneously show high activity at low temperatures and thermostability by ASR. The thermal stability of both ancestral IPMDHs designed in this study was slightly greater than that of the thermophilic IPMDH. High thermal stabilities have been similarly observed in many other reconstructed ancestral proteins^29,55–57,72^. Moreover, the ancestral enzymes had enhanced low-temperature activities relative to the thermophilic enzyme. Therefore, our current study exemplifies how the reconstruction of ancestral sequence can serve as an effective methodology to create thermally stable enzymes with higher low-temperature activity as compared with a thermophilic enzyme although their activities are lower than those of their mesophilic homologues.

The difference in thermal stability and magnitude of catalytic activity between thermophilic and mesophilic proteins has sometimes been argued on the basis of protein topology and oligomerization state. However, change in protein topology is not the case for IPMDH because all of IPMDHs with known tertiary structure share the same protein topology. Although homotetrameric structures are observed in some hyperthermophilic IPMDH, many naturally-occurring IPMDHs self-associate as dimeric structures. Analytical gel filtration suggested that ancIPMDH-IQ and ancIPMDH-ML exist purely or predominantly as homodimers (Fig. 3), a quaternary structure found for most extant IPMDHs. Therefore, the oligomerization state seems not to be responsible for the thermal stability and catalytic properties of the ancestral enzymes. The difference in thermal stability between thermophilic and mesophilic proteins has also been argued on the basis of their amino acid compositions. It has been also shown that the unfolding temperature of a protein reflects the habitat temperature of its host^56,73^. By comparing the amino acid sequences of a set of well-conserved proteins, Shakhnovich and coworkers found that the total content of Ile, Val, Tyr, Trp, Arg, Glu and Leu correlates well with the habitat temperature of organisms^74^. Accordingly, the content of these seven types of amino acids (%IVYWREL) in a protein is thought to correlate with its thermal stability. We therefore calculated the %IVYWREL values of the ancestral and extant IPMDHs (Table S1), which showed that the thermophilic *T. thermophilus* IPMDH has a higher %IVYWREL value (42.9%) as compared with the mesophilic IPMDHs (*B. subtilis* IPMDH, 40.3%; *S. cerevisiae* IPMDH, 37.2%). The %IVYWREL values of the psychrophilic *S. violacea*, *P. haloplanktis* and *P. cryohalolentis* IPMDHs are 39.0%, 36.0% and 38.8%, respectively. Thus, this correlation roughly holds for the extant IPMDHs. The %IVYWREL values of ancIPMDH-IQ (45.3%) and ancIPMDH-ML (44.4%) were greater than that of *T. thermophilus* IPMDH, which is consistent with their experimentally determined *T*_m_ values being higher than that of the thermophilic enzyme.

In large part, thermally stable enzymes are generally less active at moderate temperatures than are their less stable homologues^41,50,65,66^. However, ancIPMDH-IQ and ancIPMDH-ML were slightly more thermally stable and more catalytically active at 25 °C as compared with the *T. thermophilus* IPMDH. It has been pointed out that the catalytic efficiency of an enzyme at low temperature correlates with the localized flexibility of the active site^50,65,75,76^. In addition, we previously showed that enhancing the volume of the coenzyme-binding pocket can improve catalytic efficiency of *T. thermophilus* IPMDH at low temperatures^44^. Because no crystal and/or solution structures of the ancestral IPMDHs are available, we explored structural factors contributing to the enhanced low-temperature activity of the ancestral enzymes by using the crystal structures of *T. thermophilus* IPMDH (PDB code: 1HEX)^77^ as a guide. Figures S3a and S3b show that 17 residues are located in the NAD^+^-binding pocket and involved in binding to the coenzyme. These 17 residues are also conserved in the ancIPMDH-IQ and ancIPMDH-ML sequences (Fig. S3c), indicating that the ancestral and thermophilic enzymes share the same side chains in the coenzyme-binding pocket. In addition, the flanking sequences of the 17 residues involved in NAD^+^ binding are relatively well conserved among *T. thermophilus* and ancestral IPMDHs, and no increase in the number of glycines is observed in these regions of the ancestral enzymes (Fig. S3c), although a higher number of glycines would increase the backbone flexibility and therefore induce improved catalytic activity at low temperature. Therefore, the enhanced low-temperature activity of the ancestral enzymes is likely to be induced by a subtle change in backbone conformation. Our steady-state kinetic experiment showed that the ancestral IPMDHs have a more unfavorable *K*_m_^NAD^ than does *T. thermophilus* IPMDH. It is possible that the ancestral IPMDHs have an expanded binding pocket for NAD^+^ that weakens the packing of the bound coenzyme, thereby improving their low-temperature catalytic activity.

Residues 277 and 278 in the ancestral IPMDHs (residues 272 and 273 in *T. thermophilus* IPMDH), which were mutated in ancIPMDH-IQ-VAHG and ancIPMDH-ML-VAHG, are located near the NAD^+^ binding pocket (Figs. S3a and S3b). The non-polar side chain of residue 277 is directly opposite to the NAD^+^ binding pocket but its main chain oxygen interacts with both bound D-3-IPM and NAD^+^. Moreover, the side chain of residue 278 directly interacts with the adenine of NAD^+^. Because the two mutated residues in the ancestral IPMDH mutants both have side chains of smaller volume than those that were replaced, the mutations plausibly expand the binding pocket for NAD^+^. In addition, substitution with a glycine would increase the flexibility of the peptide backbone and therefore contribute to the improved low-temperature activity and slightly worsened thermal stability.

The remaining important question involves how the ancestral sequences behave differently from the extant sequences and confer the observed differences in behavior. However, based on the experimental data provided herein, it is difficult to predict which specific interactions contribute to the smaller *E*_a_ values and high *T*_m_ values. Therefore, further structural and mechanistic analyses, including docking and molecular dynamics simulations, and even quantum mechanics*/*molecular mechanics methods, are necessary to reveal how ancestral enzymes achieved great thermal stability and mesophilic enzyme-like catalytic properties.

In conclusion, reconstruction of ancestral sequences led to the production of thermally stable IPMDHs whose unfolding midpoint temperatures were approximately 90 °C, as reported in previous studies^31,55–57^. In addition, the ancestral enzymes had catalytic properties similar to those of mesophilic enzymes—namely, a smaller *E*_a_ for the catalytic reaction, a larger *K*_m_ for the coenzyme, higher specific activity, and higher catalytic turnover at low temperature^49–51^. Therefore, the present data show that ASR may be a way to create thermally stable enzymes whose catalytic properties are better adapted to low reaction temperatures. Thus, the present work provides another example of the utility of ASR. However, the *T*_m_ of ancIPMDH-IQ and ancIPMDH-ML (88 and 90 °C) were still lower than that of IPMDH from the hyperthermophilic archaeon *Sulfolobus tokodaii* (96 °C)^38^, although the specific activities of the ancestral enzymes (4.3 and 2.9 U/mg) were much greater than that of the hyperthermophilic enzyme at 30 °C (0.88 U/mg). In addition, the catalytic efficiency of the ancestral enzymes remained lower than those of the mesophilic IPMDHs below 50 °C. An ancestral IPMDH corresponding to the last common ancestor of *Bacillus* species, which has been reconstructed by others^45^, had a *T*_m_ value of 65.3 °C, which is approximately 25 °C lower than those of ancIPMDH-IQ and ancIPMDH-ML, although its catalytic turnover at 70 °C was higher than those of our ancestral enzymes. Therefore, reconstruction of an ancestral IPMDH corresponding to another node of the tree used to infer ancIPMDH-IQ and ancIPMDH-ML sequences is likely to create enzymes that have a different balance of thermal stability and catalytic efficiency. Moreover, the application of a directed evolution technique^18,19^ to the ancestral IPMDHs designed in this study would help to further improve their thermal stability and catalytic efficiency at low temperature. Such studies will be the focus of future research.

## Materials and methods

### Phylogenetic analysis and ancestral sequence inference

Generally, ASR consists of the following steps. Homologous amino acid sequences of the target protein are first collected from databases and subjected to multiple sequence alignment. A phylogenetic tree is then built and the amino acid sequence of an ancestral node is inferred. The next step is reconstruction of the inferred ancestral amino acid sequence by genetic engineering: the inferred sequence is reverse-translated into a nucleotide sequence, and the resulting gene is artificially synthesized. Lastly, the inferred amino acid sequence is synthesized by expressing the gene in a host organism such as *E. coli*.

In this study, a total of 727 species, 591 bacteria and 136 archaea, were selected from species whose genome sequences were publicly available. The 591 bacterial species included 45 thermophiles, 112 mesophiles and 4 psychrophiles. The remaining 430 species were found in environmental samples and have not yet been cultured and therefore their actual growth temperatures are unknown. In general, we selected species that have been well studied because they are likely to have a high degree of genomic sequence accuracy. The names and taxonomic classification of the selected species are listed in the Supplementary Dataset. Amino acid sequence data for the selected organisms were downloaded from the National Center for Biotechnology Information (NCBI, https://www.ncbi.nlm.nih.gov/) to construct an in-house database comprising all protein sequences from the 727 organisms. This in-house database was used for subsequent BLAST searches.

The amino acid sequences of 3-isopropylmalate dehydrogenase (IPMDH), homoisocitrate dehydrogenase (HICDH), and isocitrate dehydrogenase (ICDH) from *T. thermophilus* (accession numbers: AAS81211.1, AAS81354.1, and AAS81514.1, respectively), two IPMDHs from *Methanococcus maripaludis* (CAF30095.1, CAF30436.1), and HICDH from *Pyrococcus horikoshii* (BAA30836.1) were downloaded from the NCBI database. Using these six sequences as query sequences, we searched for homologous amino acid sequences in the aforementioned in-house database. As a result, 913 bacterial and 291 archaeal sequences were collected. After removing duplicate sequences, the remaining 729 sequences were aligned by using MAFFT ver.7.3^78^. Next, sequences that differed significantly from the others in length were excluded and the remaining 594 sequences (435 bacterial and 159 archaeal sequences) were re-aligned by MAFFT, incorporating secondary structural information of IPMDH, HICDH and ICDH (PDBID: 1WPW, 1OSI, 1X0L, 5HN3, 2D1C, 2DHT, and 1ZOR), and then manually corrected to generate the multiple sequence alignment that was subsequently used for tree building.

Well-aligned regions were selected from the final alignment by using the automated1 mode of trimAl^79^. IQ-TREE ver.1.6.9^46^, in conjunction with the LG + R10 amino acid substitution model, was used to build the phylogenetic tree. The LG + R10 model was selected as the optimal amino acid substitution model by ModelFinder^80^. From the resulting phylogenetic tree, we inferred ancestral sequences by using IQ-TREE with the LG + R10 model and CodeML in PAML^47^ with the LG + Gamma (eight-class) model. We also used GASP^81^ to estimate the location of gaps in the ancestral sequences. The ancestral sequence predicted by IQ-TREE was named ancIPMDH-IQ, and that predicted by CodeML was named ancIPMDH-ML.

### Construction of expression plasmids for ancestral and extant mesophilic IPMDHs

The inferred amino acid sequences of the ancestral IPMDHs were reverse-translated to generate nucleotide sequences encoding the ancestral amino acid sequences. The codon usage was optimized for an *E. coli* expression system. The nucleotide sequences were artificially synthesized by Eurofins Genomics (Tokyo) and then cloned into the *Nde*I-*Bam*HI site of plasmid pET23a(+) (Merck, Tokyo). For comparison, mesophilic IPMDHs from *B. subtilis* and *S. cerevisiae* were prepared and characterized. The respective genes were cloned into the *Nde*I-*Bam*HI site of plasmid pET21c(+) (Merck, Tokyo) and expressed in *E. coli*.

### Site-directed mutagenesis

Genes encoding ancestral IPMDH mutants containing Val277 → Ala and His278 → Gly substitutions were amplified by using the splicing-by-overlap-extension PCR method^82^. To amplify the mutagenized genes, the PCR reaction solution contained 1 × PCR buffer for KOD-plus DNA polymerization, 1 mM MgSO_4_, dNTPs at 0.2 mM each, synthetic oligonucleotides at 0.4 μM each, 1.0 unit of KOD-plus DNA polymerase (Toyobo, Osaka), and 0.2 ng/μl of template DNA. The oligonucleotides used were 5′-GCG CTG ACC CAC CAG CCG GTT CAT ATA AG-3′ and 5′-CTT ATA TGA ACC GGC TGG GTC AGC GC-3′ for the mutagenesis of ancIPMDH-IQ, and 5′-GCG CAC TGC CAC CAG CGG GCT CAT AG-3′ and 5′-CTA TGA GCC CGC TGG CAG TGC GC-3′ for the mutagenesis of ancIPMDH-ML. The following time–temperature program was used: step 1, 95 °C, 3 min; step 2, 95 °C, 30 s; step 3, 55 °C, 30 s; step 4, 68 °C, 1 min; and steps 2–4 were repeated 25 times. The amplified DNAs were then digested with *Nd*eI and *Bam*HI (New England Biolabs, Tokyo) and cloned into the *Nde*I–*Bam*HI site of pET23a( +) (Merck, Tokyo).

### Enzyme purification

To produce the ancestral and extant mesophilic IPMDHs, *E. coli* Rosetta2 (DE3) was transformed with the respective expression plasmids. Each transformant was cultured in Luria–Bertani medium supplemented with 150 μg/mL of ampicillin. Overnight Express Autoinduction system 1 reagent (Merck, Tokyo) was used to induce gene expression. This induction system allows the induction of protein expression without the need to monitor cell density during culturing and without adding a conventional induction reagent such as isopropyl β-D-1-thiogalactopyranoside. After cultivation overnight at 37 °C (ancestral IPMDHs) or 30 °C (mesophilic IPMDHs), the cells were harvested by centrifugation, and then disrupted by sonication followed by centrifugation at 60,000 × *g* for 20 min. To purify the ancestral IPMDHs, the supernatants were individually heat-treated at 70 °C for 20 min to precipitate proteins originating from *E. coli*. After centrifugation again at 60,000 × *g* for 20 min, the supernatants were successively passed through HiTrap-Butyl and ResourceQ (Cytiva, Tokyo). To purify the mesophilic IPMDHs, the soluble fractions of the cell lysates were successively passed through HiTrap-Q, HiTrap-Butyl and ResourceQ (Cytiva, Tokyo). The purity of the enzymes used in this study was > 95% as judged by the results of SDS–polyacrylamide gel electrophoresis coupled with Coomassie Blue staining.

The concentrations of protein solutions were determined from the OD_280_ values as described by Pace and colleagues^83^, who modified the procedure described by Gill and von Hippel^84^.

### Analytical gel filtration

The oligomeric state of the ancestral IPMDHs was determined by analytical gel filtration using a Superdex200 Increase column (Cytiva, Tokyo). Aliquots of protein (0.1 ml) were loaded onto the column and elution was performed at 25 °C with a flow rate of 0.7 ml/min and 20 mM potassium phosphate, pH 7.6, 150 mM KCl, 1 mM EDTA. The eluent was monitored by absorbance at 280 nm.

### Thermal stability measurement

CD measurements were carried out using a J-1100 spectropolarimeter (Jasco, Hachioji). The enzymes were dissolved in 20 mM potassium phosphate buffer (pH 7.6), 1 mM EDTA at a concentration of 5 μM and placed in a sample cell with a 0.1-cm path length. The temperature of the enzyme solution was increased at a rate of 1.0 °C/min by using a programmable temperature controller. Thermal denaturation was observed by monitoring the change in ellipticity at 222 nm, which reflects the abundance of α-helices in a protein.

### Enzyme activity measurements

Specific activity was measured at various temperatures in an assay solution comprised of 50 mM HEPES (pH 8.0), 100 mM KCl, 5 mM MgCl_2_, 0.2 mM D-3-IPM and 5.0 mM NAD^+^ by monitoring the increase in absorbance at 340 nm, which reflects the generation of NADH, a product of the reaction catalyzed by IPMDH. One enzyme unit was defined as the formation of 1 μmol of NADH per min.

Michaelis constant (*K*_m_) values for D-3-IPM, the substrate of IPMDH, were determined in steady-state experiments with an assay solution containing 50 mM HEPES (pH 8.0), 100 mM KCl, 5 mM MgCl_2_, 5 mM NAD^+^, and various concentrations of D-3-IPM. To determine the values of *K*_m_ for the coenzyme NAD^+^ and *k*_cat_, the concentration of NAD^+^ was varied while the D-3-IPM concentration was fixed (0.2 mM). Protein concentrations were 20 nM (25 °C), 6.0 nM (40 °C) or 2.0 nM (70 °C). To obtain the kinetic parameters, the observed steady-state velocity data were fitted to the Michaelis–Menten equation by using the Enzyme Kinetics module of SigmaPlot version 13.0, from Systat Software, Inc., San Jose California USA, www.systatsoftware.com.

## Supplementary information

Supplementary Information

Supplementary Dataset

Supplementary Figure S1.

## Data Availability

The data that support the findings of this study are available from the corresponding author upon reasonable request.
