# Supplementary material for: Ancestral sequence reconstruction produces thermally stable enzymes with mesophilic enzyme-like catalytic properties
Source: Sci Rep. 2020 Sep 23;10:15493. doi: 10.1038/s41598-020-72418-4 (PMC7511310; doi:10.1038/s41598-020-72418-4)
Supplement: Supplementary file 1 — Supplementary Information [file 41598_2020_72418_MOESM1_ESM.pdf]

Supplementary materials

Ancestral sequence reconstruction produces thermally stable enzymes with mesophilic enzyme-like catalytic properties

Ryutaro Furukawa[a], Wakako Toma[a], Koji Yamazaki[a], Satoshi Akanuma[a,1]

[a]Faculty of Human Sciences, Waseda University, 2-579-15 Mikajima, Tokorozawa, Saitama 359-1192, Japan

[1]Corresponding author: Faculty of Human Sciences, Waseda University, 2-579-15 Mikajima, Tokorozawa, Saitama 359-1192, Japan.

Tel: +81-4-2947-6727 E-mail: akanuma@waseda.jp

Table S1. Amino acid composition of the ancestral and extant IPMDHs.

	ancIPMDH-IQ	ancIPMDH-ML	*Tt*IPMDH [a]	*Bs*IPMDH [b]	*Sc*IPMDH [c]
Ala	40	41	43	38	37
Cys	2	2	0	1	2
Asp	18	19	15	20	23
Glu	42	37	33	37	23
Phe	9	9	14	16	10
Gly	31	31	36	25	29
His	2	2	6	6	6
Ile	17	18	11	17	25
Lys	21	19	16	22	28
Leu	36	36	38	38	40
Met	12	12	6	10	6
Asn	8	8	5	15	14
Pro	19	19	25	15	23
Gln	6	8	2	8	9
Arg	18	20	23	18	11
Ser	15	16	16	28	25
Thr	13	13	13	14	16
Val	37	37	35	29	28
Trp	2	2	3	2	4
Tyr	10	9	5	6	4
**%IVYWREL [d]**	**45.3**	**44.4**	**42.9**	**40.3**	**37.2**

[a] *Tt*IPMDH, *T. thermophilus* IPMDH.

[b] *Bs*IPMDH, *B. subtilis* IPMDH.

[c] *Sc*IPMDH, *S. cerevisiae* IPMDH.

[d] %IVYWREL, content of seven amino acid types (Ile, Val, Tyr, Trp, Arg, Glu, Leu).

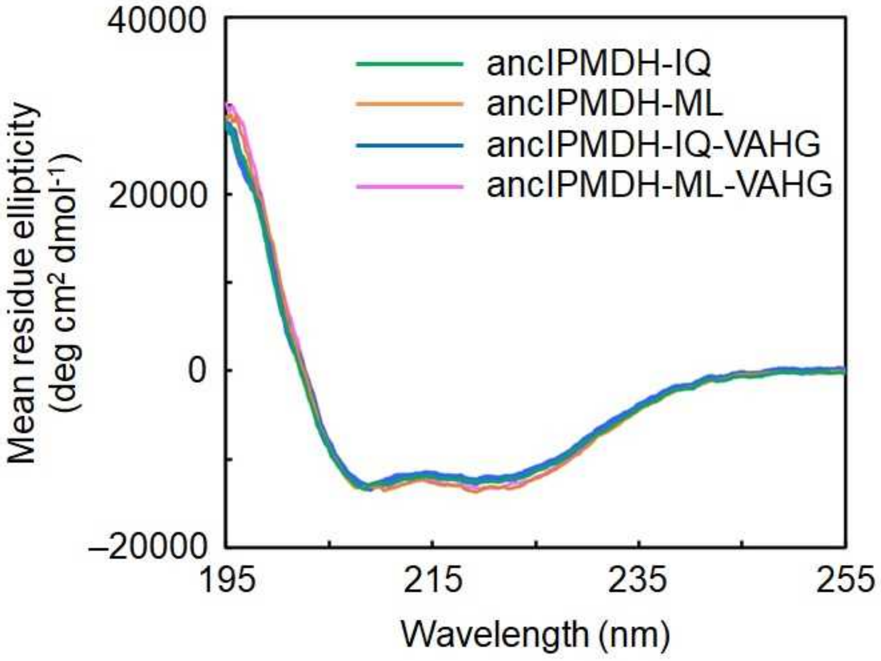

Fig. S2. Far-UV CD spectra of ancIPMDH-IQ, ancIPMDH-ML, ancIPMDH-IQ-VAHG and ancIPMDH-ML-VAHG. The proteins were 10 μM in 20 mM Tris-HCl, pH 7.5, 1 mM EDTA. The temperature was 25°C, and a 0.1 cm path-length cell was used.

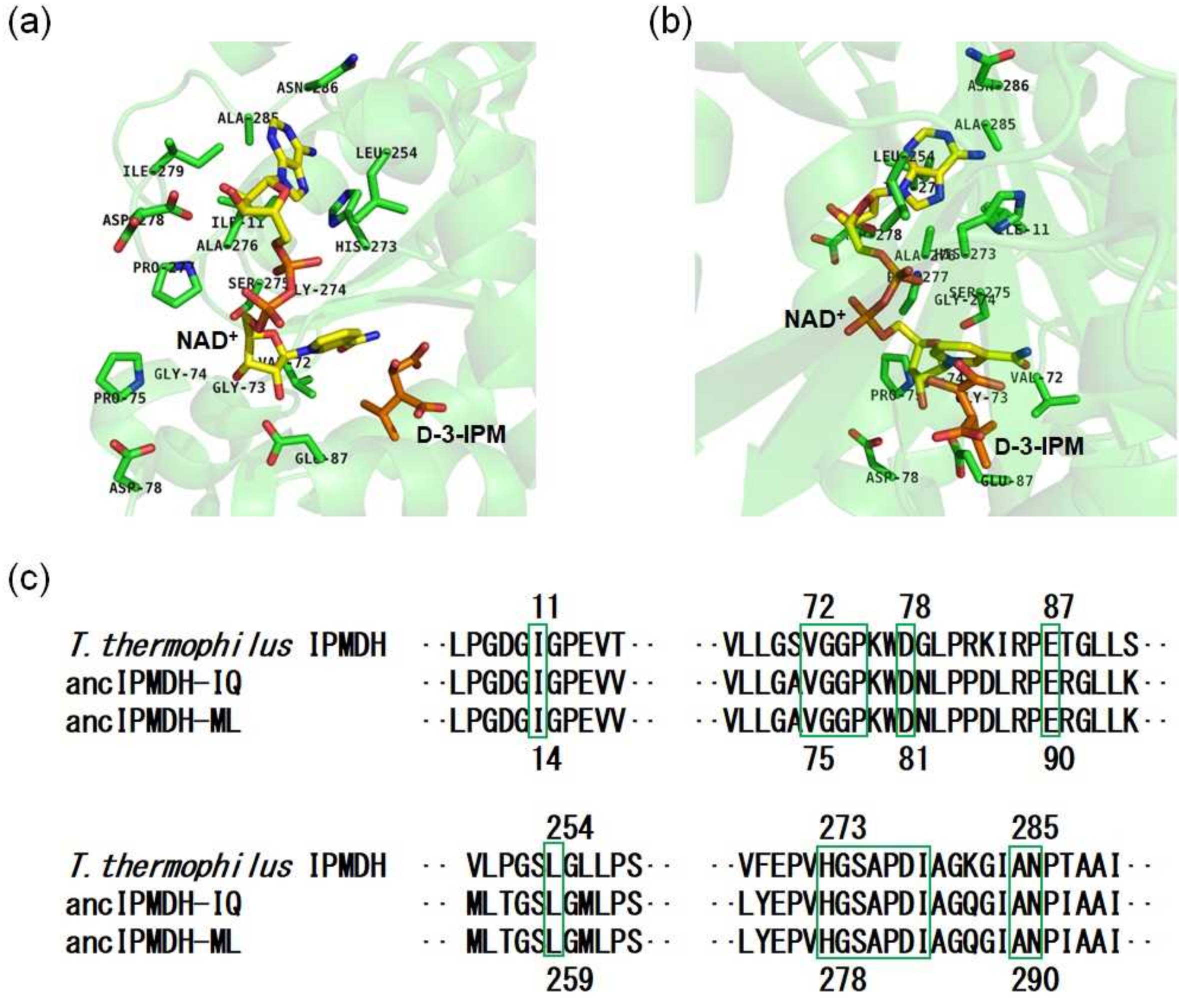

Fig. S3. Residues involved in NAD$^+$ binding. (**a**) The NAD$^+$-binding pocket of *T. thermophilus* IPMDH with NAD$^+$ and D-3-IPM bound in the pocket. The side chains of the 17 residues that seem to be involved in NAD$^+$ binding are shown as sticks and labeled. The bound NAD$^+$ and D-3-IPM are also shown as sticks and labeled. The image was drawn with PyMOL (http://www.pymol.org). (**b**) View in (a) rotated 45° left. (**c**) Comparison of the NAD$^+$-binding residues and their flanking sequences of *T. thermophilus* IPMDH with those of the ancestral IPMDHs. Numbers above and below the sequences are those of *T. thermophilus* and ancestral IPMDHs, respectively. The NAD$^+$-binding residues are boxed.

Legend to Fig. S1

Phylogenetic trees used to infer the amino acid sequences of ancestral IPMDHs. Organism names and database accession numbers are indicated. The color code is the same as in Fig. 1. The number on each node shows the RELL bootstrap probability.

Supplementary dataset

The supplementary dataset summarizes the accession numbers for the amino acid sequences of IPMDH and its evolutionarily related proteins used to build the phylogenetic tree. Non-colored accession numbers were removed from the alignment during the process of final inference of the phylogenetic tree.
