# Supplementary figures and images for: Ancestral sequence reconstruction produces thermally stable enzymes with mesophilic enzyme-like catalytic properties
Source: Sci Rep. 2020 Sep 23;10:15493. doi: 10.1038/s41598-020-72418-4 (PMC7511310; doi:10.1038/s41598-020-72418-4)

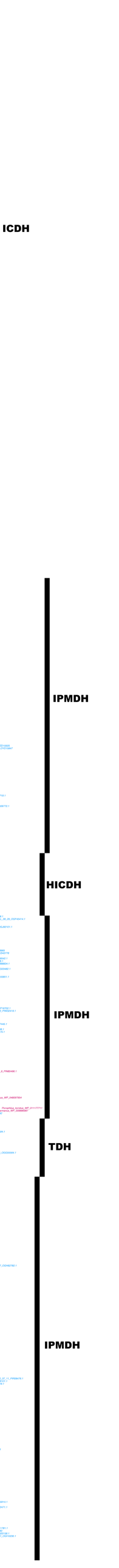

Supplement: Supplementary file 3 — Supplementary Figure S1. [file 41598_2020_72418_MOESM3_ESM.pdf]
